# Evaluation of the QIAstat-Dx Respiratory SARS-CoV-2 Panel, the First Rapid Multiplex PCR Commercial Assay for SARS-CoV-2 Detection

**DOI:** 10.1128/JCM.00630-20

**Published:** 2020-07-23

**Authors:** Benoit Visseaux, Quentin Le Hingrat, Gilles Collin, Donia Bouzid, Samuel Lebourgeois, Diane Le Pluart, Laurène Deconinck, François-Xavier Lescure, Jean-Christophe Lucet, Lila Bouadma, Jean-François Timsit, Diane Descamps, Yazdan Yazdanpanah, Enrique Casalino, Nadhira Houhou-Fidouh

**Affiliations:** a Université de Paris, Assistance Publique – Hôpitaux de Paris, Service de Virologie, Hôpital Bichat, Paris, France; b UMR 1137-IAME, Decision Sciences in Infectious Diseases Control and Care (DeSCID), INSERM, Université de Paris, Paris, France; c Université de Paris, Assistance Publique – Hôpitaux de Paris, Service d’Accueil des Urgences, Hôpital Bichat, Paris, France; d Université de Paris, Service de Maladies Infectieuses et Tropicales, Hôpital Bichat Claude Bernard, Assistance Publique – Hôpitaux de Paris, Paris, France; e Université de Paris, Unité d’Hygiène et de Lutte contre les Infections Nosocomiales, Hôpital Bichat Claude Bernard, Assistance Publique – Hôpitaux de Paris, Paris, France; f Université de Paris, Réanimation Médicale et Infectieuse, Hôpital Bichat Claude Bernard, Assistance Publique – Hôpitaux de Paris, Paris, France; Boston Children’s Hospital

**Keywords:** COVID-19, SARS-CoV-2, diagnostics, mPCR, rapid tests

## Abstract

In the race to contain severe acute respiratory syndrome coronavirus 2 (SARS-CoV-2), efficient detection and triage of infected patients must rely on rapid and reliable testing. In this work, we performed the first evaluation of the QIAstat-Dx respiratory SARS-CoV-2 panel (QIAstat-SARS) for SARS-CoV-2 detection. This assay is the first rapid multiplex PCR (mPCR) assay, including SARS-CoV-2 detection, and is fully compatible with a non-PCR-trained laboratory or point-of-care (PoC) testing. This evaluation was performed using 69 primary clinical samples (66 nasopharyngeal swabs [NPS], 1 bronchoalveolar lavage fluid sample [BAL], 1 tracheal aspirate sample, and 1 bronchial aspirate sample) comparing SARS-CoV-2 detection with the currently WHO-recommended reverse transcription-PCR (RT-PCR) (WHO-RT-PCR) workflow. Additionally, a comparative limit of detection (LoD) assessment was performed for QIAstat-SARS and WHO-RT-PCR using a quantified clinical sample. Compatibility of sample pretreatment for viral neutralization or viscous samples with the QIAstat-SARS system were also tested. The QIAstat-Dx respiratory SARS-CoV-2 panel demonstrated a sensitivity comparable to that of the WHO-recommended assay with a limit of detection at 1,000 copies/ml. The overall percent agreement between QIAstat-Dx SARS and WHO-RT-PCR on 69 clinical samples was 97% with a sensitivity of 100% (40/40) and specificity at 93% (27/29). No cross-reaction was encountered for any other respiratory viruses or bacteria included in the panel. The QIAstat-SARS rapid multiplex PCR panel provides a highly sensitive, robust, and accurate assay for rapid detection of SARS-CoV-2. This assay allows rapid decisions even in non-PCR-trained laboratory or point-of-care testing, allowing innovative organization.

## Abstract (precis)

In the race to contain severe acute respiratory syndrome coronavirus 2 (SARS-CoV-2), efficient detection and triage of infected patients must rely on rapid and reliable testing. In this work, we performed the first evaluation of the QIAstat-Dx respiratory SARS-CoV-2 panel (QIAstat-SARS) for SARS-CoV-2 detection. This assay is the first rapid multiplex PCR (mPCR) assay, including SARS-CoV-2 detection, and is fully compatible with a non-PCR-trained laboratory or point-of-care (PoC) testing. This evaluation was performed using 69 primary clinical samples (66 nasopharyngeal swabs [NPS], 1 bronchoalveolar lavage fluid sample [BAL], 1 tracheal aspirate sample, and 1 bronchial aspirate sample) comparing SARS-CoV-2 detection with the currently WHO-recommended reverse transcription-PCR (RT-PCR) (WHO-RT-PCR) workflow.

## INTRODUCTION

Since the novel coronavirus was identified in China in December 2019, it has spread globally and has been declared a pandemic by the WHO on 11 March 2020 (1). The etiological agent of the infection is the severe acute respiratory syndrome coronavirus 2 (SARS-CoV-2) (2). The availability and reliability of rapid assays allowing quick identification of infected individuals are the cornerstone for isolation, patient management, and prevention measures. In-house and commercial reverse transcription-PCR (RT-PCR) assays have been quickly developed (3). The QIAstat-Dx respiratory SARS-CoV-2 panel (QIAstat-Dx SARS) is a CE-IVD (*in vitro* diagnostic) product based on the previously CE-marked QIAstat-Dx respiratory panel, a qualitative multiplexed nucleic acid-based test detecting 21 viruses and bacteria, recently developed and demonstrating performance similar to that of FilmArray RP1.7 (bioMérieux, BioFire) or Anyplex RV16 (Seegene) for human respiratory viruses (4, 5). The QIAstat-Dx cartridge allows the loading of the nasopharyngeal swab (NPS) resuspended in transport medium through the liquid port or the direct insertion of the NPS into the cartridge without additional manipulation. This innovative design allows sample testing on demand in any laboratory, even in a non-PCR-trained laboratory, or as a point-of-care (PoC) test with minimal training. The QIAstat-Dx SARS version of the panel adds the detection of SARS-CoV-2 in a previously unused amplification chamber, without any change for sampling, extraction, and PCR conditions for detection of other respiratory viruses (4, 5). As for a few other rapid PCR assays available thus far, results are provided in about 1 h, compared to the labor-intensive 3 to 4 h in the laboratory workflow of most in-house PCR assays. The comprehensiveness and versatility of the kit can potentially provide a useful tool, allowing innovative organization to test patients suspected of having coronavirus 2019 (COVID-19) rapidly and with discrimination of other respiratory pathogens. This is of importance to accelerate isolation, patient management, and treatment decisions.

Here, we report the first independent validation of this new commercial PCR assay for SARS-CoV-2 detection, using clinical samples both in laboratory and in the emergency department as a point-of-care test.

## MATERIALS AND METHODS

### Analytical sensitivity analysis of the QIAstat-Dx respiratory SARS-CoV-2 panel.

A preliminary sensitivity analysis was conducted using a 1:4 dilution of a SARS-CoV-2-positive nasopharyngeal sample (sample 1), presenting initial threshold cycle (*C_T_*) values of 19.2 and 21.1 for the E and RdRp gene with the currently WHO-recommended RT-PCR assay (WHO-RT-PCR), respectively. This sample was then serially diluted 1:10 and tested with both QIAstat-Dx SARS and WHO-RT-PCR assays (3). To further assess the comparative limit of detection (LoD) between the two methods, a second sample was quantified using a standard curve of synthetic DNA of 300 nucleotides corresponding to the amplified E gene region. A RNA transcript control, obtained from the European Virus Archive Program, was added as a positive control. The limit of detection (LoD) was determined by testing multiple replicates of serial twofold dilutions of the quantified sample. As recommended by the European Network of GMO Laboratories for LoD definition (6), an initial dilution below the expected LoD dilution was tested, to increase the concentration until obtaining 10 out of 10 positive replicates (10/10). All dilutions were done using Virocult transport medium and kept at +4°C before testing within 24 h.

Briefly, for the WHO-RT-PCR, RNA was extracted from 200 μl of clinical samples with the EZ1 virus minikit v2.0 (Qiagen) and eluted in 60 μl. RT-PCR targeting E and Orf1 genes were performed as described by Corman et al. (3). The QIAstat-SARS targets the same E gene region as the WHO-RT-PCR and a different region located on the Orf1 gene, both reported with the same fluorophore in a single dedicated reaction chamber.

### Evaluation of specificity and pretreatment conditions.

To assess the specificity of the QIAstat-Dx SARS assay toward other coronaviruses and various respiratory microorganisms included in the panel, each target was assessed using pools of three positive samples obtained during the last 6 months. An evaluation of pretreatment conditions was conducted. Various conditions were tested as follows: a prelysis step with AVL buffer (Qiagen), using a 4:1 ratio of AVL buffer and NPS sample; heating, 20 min at 65°C, used for viral safety (7); and proteinase K, used 1:10 at 20 mg/ml to reduce the viscosity of induced sputum.

### Performance comparison using clinical samples.

We included 66 nasopharyngeal swab (NPS) specimens from patients hospitalized in the Bichat Claude Bernard teaching hospital, Paris, France. Additionally, three lower respiratory samples (the earliest positive bronchoalveolar lavage [BAL] fluid sample, tracheal aspirate sample, and bronchial aspirate sample from different patients available in our laboratory) were tested to evaluate alternative off-label samples. The 69 samples tested with QIAstat-Dx were compared to the standard-of-care diagnostic method, the WHO-RT-PCR. The 3 lower respiratory samples and 23 out of the 66 tested NPS samples were used to assess the liquid sample workflow. These specimens were taken from patients suspected of having COVID-19, collected in Virocult viral transport medium (Sigma), and tested in the laboratory by adding 300 μl into the liquid port of the cartridge, as recommended (central-lab samples). The remaining 42 NPS samples were collected from 42 patients suspected of having COVID-19 and tested in the emergency department. One NPS specimen was directly introduced into the swab port of the cartridge, as recommended by the manufacturer for point-of-care testing (POC samples), and as for any implementation of new PoC testing, a second nasopharyngeal swab was collected in Virocult viral transport medium and assessed in a central laboratory using the WHO-RT-PCR workflow. In case of discrepancies between the QIAstat-Dx SARS and the WHO-RT-PCR, a third method was performed (Cobas SARS-CoV-2 test; Roche). The result of the third method was not used for QIAstat-Dx SARS performance calculation. The use of respiratory viruses’ multiplex PCR (mPCR) PoC testing data for research purposes was approved by the Bichat Claude Bernard ethic committee (N2019-050). All hospitalized patients, included those whose samples were used for laboratory testing, were included in the national French-COVID19 cohort, and written consent was obtained for clinical and biological substudies.

## RESULTS

### Analytical sensitivity analysis of the QIAstat-Dx respiratory SARS-CoV-2 panel.

All results obtained from 1:10 serial dilutions of sample 1 are depicted in Table 1. The virus was detected for all dilutions up to 1:40,000 by both QIAstat-Dx SARS and WHO-RT-PCR methods. At 1:400,000 dilution, the virus was detected only in 5/5 and 3/5 replicates by WHO-RT-PCR and QIAstat-SARS, respectively. Regarding the LoD assessment, all the results obtained with the serial 1:2 dilutions of sample 2 around the expected LoD are depicted in Table 2. The detection of 10/10 replicates by the QIAstat-Dx SARS was achieved for a viral load estimated at 1,000 copies/ml, i.e., for an input of 300 copies in the cartridge for QIAstat-SARS and of 16 copies/PCR for WHO-RT-PCR under our conditions.

**TABLE 1:** Initial sensitivity assessment of QIAstat-Dx respiratory 2019-nCoV panel (Qiagen) and the WHO-recommended Charité RT-PCR assay for the E gene and RdRp gene (4) on serial dilutions of a positive nasopharyngeal clinical sample (strain 1)

Dilution	QIAstat-Dx		E gene		RdRp gene	
	*C_T_* value	Interpretation	*C_T_* value	Interpretation	*C_T_* value	Interpretation
1/4	21.1	Positive	21.5	Positive	23.0	Positive
1/40	25.7	Positive	25.6	Positive	26.1	Positive
1/400	28.0	Positive	29.6	Positive	31.3	Positive
1/4,000	31.0	Positive	33.1	Positive	Not detected	Negative
1/40,000	34.8	Positive	35.7	Positive	Not detected	Negative
	35.2	Positive	36.0	Positive	Not detected	Negative
	35.3	Positive	36.2	Positive	Not detected	Negative
1/400,000	36.3	Positive	37.3	Positive	Not detected	Negative
	37.1	Positive	37.6	Positive	Not detected	Negative
	37.4	Positive	38.4	Positive	Not detected	Negative
	Not detected	Negative	38.5	Positive	Not detected	Negative
	Not detected	Negative	38.7	Positive	Not detected	Negative
1/4,000,000	35.9	Positive	38.4	Positive	Not detected	Negative
	Not detected	Negative	40.0	Negative	Not detected	Negative
	Not detected	Negative	40.4	Negative	Not detected	Negative
	Not detected	Negative	Not detected	Negative	Not detected	Negative
	Not detected	Negative	Not detected	Negative	Not detected	Negative

**TABLE 2:** Determination of the limit of detection of the QIAstat-Dx assay and comparison with the WHO-recommended PCR assay^*a*^

Viral load and replicate^*b*^	QIAstat-Dx	E gene	RdRp gene
2000 cp/ml			
Replicate 1	35.0	35.4	Negative
Replicate 2	35.6	35.4	Negative
Replicate 3	35.7	35.4	Negative
1,000 cp/ml			
Replicate 1	35.1	33.6	Negative
Replicate 2	35.1	34.5	Negative
Replicate 3	35.1	34.7	Negative
Replicate 4	35.2	34.7	Negative
Replicate 5	35.4	35.0	Negative
Replicate 6	35.7	35.2	Negative
Replicate 7	35.7	35.9	Negative
Replicate 8	35.9	35.9	Negative
Replicate 9	36.7	36.2	Negative
Replicate 10	37.6	Negative	Negative
500 cp/ml			
Replicate 1	36.8	34.3	Negative
Replicate 2	37.3	34.7	Negative
Replicate 3	37.6	36.4	Negative
Replicate 4	Negative	37.2	Negative
Replicate 5	Negative	Negative	Negative
250 cp/ml			
Replicate 1	37.8	Negative	
Replicate 2	Negative	Negative	
125 cp/ml			
Replicate 1	Negative	37.2	
Replicate 2	Negative	Negative	

a The limit of detection (LoD) is defined, according to the European Network of GMO Laboratories recommandations, as the lowest content of genetic material able to be detected in all 10 replicates. QIAstat-Dx and the WHO-recommended Charité RT-PCR assay for the E gene and RdRp gene were compared. QIAstat-Dx was tested using twofold dilutions of a second positive nasopharyngeal clinical sample (strain 2) around the previously estimated LoD until obtaining 10 replicates or a failed result. The in-house assay was tested for obtained LoD values using the same viral dilution conserved at +4°C, in the same 24 h.

b The estimated viral load (in copies per milliliter [cp/ml]) and replicate number are shown.

### Evaluation of specificity and pretreatment conditions.

No cross-reaction was detected either for human coronaviruses (229E, OC43, NL63, and HKU1, *n* = 2 pools of three samples for each target) or for other respiratory viruses (influenza A:H1N1 virus, A:H3N2 virus, and B virus, respiratory syncytial virus, rhinovirus, parainfluenza virus 1 and 4, and human metapneumovirus; *n* = 1 pool for each) and the bacteria included (Mycoplasma pneumoniae, Legionella pneumophila, and Bordetella pertussis, one positive sample for each). All the corresponding targets have been correctly identified in those assays. For AVL prelysis, no significant difference was observed with a nontreated sample (29.9 *C_T_* without prelysis and 30.4 *C_T_* with prelysis), and this was also true for proteinase K treatment (25.5 versus 25.5 *C_T_*).

### Performance comparison using clinical samples.

**FIG 1:**
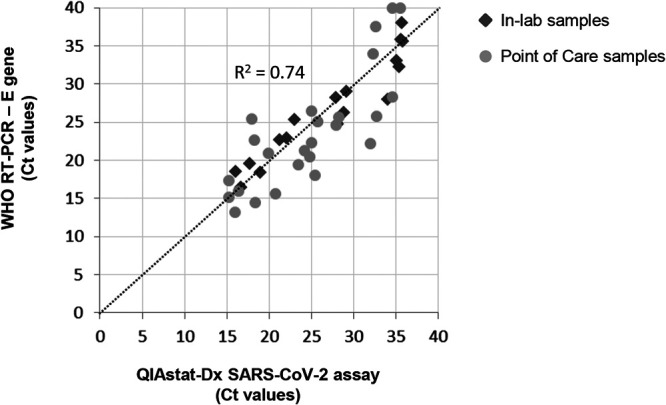
Concordance of cycle threshold values obtained with the QIAstat-Dx assay and the E gene from the WHO-recommended RT-PCR. Samples compared using the liquid sample workflow in the central laboratory (In-lab samples) are indicated by dark gray diamonds, and samples compared using the swab workflow in the emergency department (Point of Care samples) (comparison performed with two swabs, one for the QIAstat-Dx and one sent to the central laboratory for the WHO assay) are indicated by light gray circles.

All results are depicted in Fig. 1. Among the central-lab samples, the QIAstat-SARS presented a sensitivity of 100% (17/17 positive samples by WHO-RT-PCR) and a specificity at 100% (9/9 negative samples by WHO-RT-PCR). The 17 positive samples included 14 NPS and the 3 lower respiratory samples. Among the PoC samples, the QIAstat-SARS presented a sensitivity of 100% (23/23 positive samples by the WHO-RT-PCR) and a specificity of 90% (18/20 negative samples by the WHO-RT-PCR). Overall, the QIAstat-SARS presented a sensitivity of 100% (40/40) and a specificity of 93% (27/29) compared with the WHO-RT-PCR. Note that for both patients identified as positive only by QIAstat-SARS, a third PCR method was added (Cobas SARS-CoV-2 test; Roche), which provided a positive result for one of the two samples.

## DISCUSSION

This is the first independent validation of an mPCR assay including SARS-CoV-2 detection. Its performance and limit of detection on clinical samples are in line with the currently WHO-recommended PCR (3). The two workflows of the QIAstat-Dx respiratory SARS-CoV-2 panel, the liquid and direct swab ports, could be assessed despite a relatively low number of samples due to the available number of cartridges available for this evaluation. The LoD of the E gene was slightly higher than that previously estimated for WHO-RT-PCR (16 copies/PCR for 5 copies/PCR initially estimated [3]) or for QIAstat-RP-nCoV (1,000 copies/ml compared to 300 copies/ml according to the manufacturer). This may be explained by the use of clinical samples in the current work instead of transcript RNA or synthetic DNA in previous estimations and different LoD estimation methods. The poor sensitivity of the WHO assay for the RdRp gene has also been reported in a recent prepublication comparing the performances of the main reference assays (8). The sensitivity and specificity of the QIAstat-Dx SARS were excellent on clinical samples. The two positive results detected with QIAstat-SARS but not WHO-RT-PCR could be explained by low viral loads and the variability observed between the two swabs performed for PoC testing and laboratory confirmation (Fig. 1).

Multiplex assays present high reagent cost and lower number of treated samples per day than high-throughput platforms. However, multiplexing of all relevant respiratory viruses in the same cartridge allows detection of other viral infections that are part of diagnostic workflows for patients suspected of having COVID-19, especially as other viruses are cocirculating in most countries presenting SARS-CoV-2 outbreaks and may be identified in association with SARS-CoV-2 (9). Moreover, the QIAstat-SARS also offers the possibility of being used in all non-PCR-trained laboratories or as a point-of-care testing where needed and with a rapid turnaround time (approximately 67 min). Point-of-care testing for respiratory viruses using mPCR has been already suggested as improving patient care with quicker and more appropriate clinical decisions (10, 11). The benefits on patient triage, isolation management, and assessment of patients around the world are even more obvious and critically important for COVID-19.
